# Self and identity in borderline personality disorder: Agency and mental time travel

**DOI:** 10.1111/jep.12769

**Published:** 2017-05-24

**Authors:** Natalie Gold, Michalis Kyratsous

**Affiliations:** ^1^ Department of Philosophy King's College London London UK; ^2^ Department of Psychological Medicine, King's College London Institute of Psychiatry, Psychology and Neuroscience London UK; ^3^ South London and Maudsley NHS Foundation Trust London UK

**Keywords:** agency, autobiographical memory, borderline personality disorder, episodic memory, mental time travel, narrative, personal identity, self, team reasoning, temporality, unstable sense of self

## Abstract

We consider how conceptions of the self and identity from the philosophical literature can help us to understand identity disturbance in borderline personality disorder (BPD). We present 3 philosophical approaches: connectedness, narrative, and agency. We show how these map on to 3 different ways in which the self can be temporally extended. The connectedness approach is dominant in philosophy, and the narrative approach has been used by psychiatry, but we argue that the lesser‐known agency approach provides a promising way to theorize some aspects of identity disturbance in BPD. It relates the 2 diagnostic criteria of identity disturbance and disinhibition and is consistent with evidence of memory deficits and altered self‐processing in BPD patients.

## 1 INTRODUCTION

Identity disturbance is the core feature of borderline personality disorder (BPD).^1^, ^2^, ^3^, ^4^, ^5^, ^6^ DSM‐5 describes 2 types of impairment of self‐functioning^7^:

1. identity: The self is impoverished, poorly developed, or there is an unstable self‐image, which is often associated with excessive self‐criticism; chronic feelings of emptiness; and dissociative states under stress,
2. self‐direction: instability in goals, aspirations, values, and career plans.

However, the classification of identity disturbance is diffuse, covering a wide range of indicators.^8^ Clinically, the notion of identity disturbance corresponds to severe difficulties in describing personal features of oneself and others, as well as problems in developing a sense of self with beliefs, interests, and life goals that are stable over time. This can take the form of extreme and polarized self‐conceptions, feelings of puzzlement about changes in the self, lack of a coherent image of self, explosive shifts into states where the perception of self is distorted and shows weak correspondence with external reality, a lack of capacity to flexibly adapt oneself to changes, rapidly changing roles and relationships, discontinuity in self‐experience, and no clear concept of self‐development. Patients identify only with their present affective states and have no sense of their continuity over time, leading Fuchs to describe the phenomenology of identity in BPD as an “atemporal mode of existing.”^9^

Philosophers also use the concept of identity, although they do so in different ways and contexts. The word “identity” can be a false friend to psychiatrists because, in the philosophical discussion of personal identity, both “person” and “identity” are technical terms. The problem of identity is the general one of what it is to persist over time. It can equally be applied to objects, the classic example being whether a lump of clay and the statue that it becomes are the same thing. A “person” is a normative notion, introduced by Locke, of a rational being who is a locus of moral accountability.^10^ Hence, the criteria for personal identity might be different from the criteria for identity of animals and inanimate objects.

“Self” is sometimes used in philosophy, but it is not defined consistently and philosophers have disagreed about whether that means that they should stop using it.^11^, ^12^, ^13^ For instance, Locke used “self” to refer to a momentary entity with subjective, phenomenological experiences^10^; many have followed him in this usage.^14^, ^15^ However, MacIntyre has used “self” to reference the unity of a life^16^ and Taylor has used “self” synonymously with “person” and “human agent.”^17^ For both MacIntyre and Taylor, a self is something that is constructed, rather than experienced. In this paper, we will use “self” as an umbrella term that does not imply any one specific usage.

We will explore different conceptions of self over time from the philosophical literature and consider their utility for understanding BPD. We present 3 philosophical approaches, connectedness, narrative, and agency and show how these map on to 3 different ways in which the self can be temporally extended. The connectedness approach is dominant in philosophy, and the narrative approach has been used by psychiatry, but we argue that the lesser‐known agency approach provides a promising way to theorize some aspects of identity disturbance in BPD.

## 2 THREE PHILOSOPHICAL APPROACHES TO IDENTITY AND SELF OVER TIME

The traditional philosophical problem of personal identity is one in metaphysics, about the persistence of persons across time: If we take a person X at time 1 and a person Y at time 2, what would make X and Y one and the same person, and under what conditions would we want to say that X had ceased to exist and a new entity Y had come into being? Or: Are X and Y one person or two? However, personal identity is also relevant for answering other philosophical questions that relate to practical concerns, such as whether and why people should care about their future pleasure or pain (*egoistic concern*); why we can attribute moral responsibility to someone for her past actions (*moral concern*); and why we think that we can compensate someone for a burden born at another time in her life, but we cannot compensate someone else for the burden she bears (*compensatory concern*). Some philosophers have argued that starting with persistence is the wrong way to address these questions about our practical concerns. Criticisms of the persistence approach have led to the development of 2 other approaches. Hence, we start with the persistence approach.

1. Persistence and connectedness

For philosophers, the traditional question of personal identity is about persistence: What continuity is necessary for my survival and what sort of changes would result in my ceasing to exist? Historically, philosophers like Plato in the *Phaedo* and Descartes in the *Meditations* thought that continued existence was due to the presence of an immaterial soul. However, contemporary debate is between psychological continuity and biological continuity views of personal identity.

Psychological continuity views, where persistence is determined by the endurance of your mental features, enjoy most popularity nowadays. They originate with Locke, who focused on continuity of memory.^10^ Contemporary philosophers have broadened the criteria to include other psychological connections such as intentions, beliefs, desires, goals, and similarity of character. The best known modern psychological continuity theory is that of Derek Parfit, who defines strong connectedness as “the holding of particular direct psychological connections” and psychological continuity as “the holding of overlapping chains of *strong* connectedness” (italics in the original), where strong connectedness remains to be cashed out fully but consists of “enough direct connections.”^18^ *
For Parfit, personal identity also requires “uniqueness,” or being the only continuer of a past self. He introduces this condition to deal with hypothetical cases where a person undergoes fission and has 2 identical future selves. So according to Parfit, I am strongly connected to myself yesterday and to myself tomorrow, given that no dramatic changes have happened. I may not be strongly connected to myself 20 years ago—I may have forgotten some of the things I did, changed some of beliefs and desires, and become a rather different character—but I am psychologically continuous in virtue of the strong connectedness that holds over smaller units of time.

The persistence question is a technical question about ontology. We might say that someone who suffers amnesia is no longer the same person because they have changed. But there are 2 different senses of being a different person that could come into play here. If X is the person who exists before the amnesia and Y is the person who exists afterwards, we might say that X has changed and become Y—she has changed some of her characteristics—but X and Y are still the same entity. This is not the change that is implicated by the persistence question. If we say that X does not persist, that is to say that the entity that was X has ceased to exist (or has died) and a new entity, Y, has come into existence. We might think of the difference between these 2 senses as the difference between being qualitatively the same and being numerically the same.^18^ Qualitative identity is the sharing of properties, so 2 different things can be qualitatively the same if they have the same characteristics, eg, 2 white billiard balls are qualitatively but not numerically the same, and qualitative identity comes in degrees depending on similarity, eg, our 2 white billiard balls are more similar to each other than to a white golf ball. However, the same thing numerically can become qualitatively different from itself in the past, eg, when a white billiard ball is painted red, and numerical identity is binary—it is either the same ball or it is not. Psychological continuity theories associate qualitative and numerical identity because, according to these theories, whether or not a person ceases to exist may depend on whether her psychology has undergone a particular type and amount of change.

Once we understand the persistence question as being about numerical rather than qualitative identity, we can see why biological continuity theories, where persistence is determined by the endurance of the biological organism, have some attraction. We might want to say that I was a foetus or that the person who has a car accident is the same entity who is now in a persistent vegetative state. If you find those statements attractive, then you are leaning towards the view that identity over time is not simply a matter of psychology. †
Alternatively, combining the biological and psychological views, one might think that there are 2 entities, a person and a human organism, each with different persistence conditions. For instance, McMahan identifies the person with the capacity for consciousness and related brain functionings.^19^ According to him, the foetus has not yet become a person and the entity in a vegetative state is no longer a person, but both are parts of the same human organism. So the organism can exist before the person comes into being and can survive after the person has died. However, since we want to extract psychological implications, we will not consider biological views here.

2 Characterization and narrative

In philosophy, the term “personal identity” usually refers to the debate about what type of continuity is necessary for numerical identity, but persistence theories do not capture what is colloquially meant when people talk about identity. For some psychiatric purposes, we require an account of the development of identity understood as a set of affiliations, which may change over time. We can ask another set of questions, about how we characterise ourselves: What features are most central to my identity? Which beliefs, values, desires, or other psychological features make someone the person she is? Someone who is having an identity crisis might recognise that she is numerically the same person as previously, but ask who she is and whether she is qualitatively the same person as previously. As Ricoeur says, being the same is different from being a self.^20^

Rousseau's *Confessions* can be seen as an early attempt to answer these questions. Amongst contemporary philosophers, Schectman has taken them up, arguing that analytic philosophy's exclusive focus on persistence is at odds with our practical concerns.^21^ For Schechtman, it is not just that the person on the street tends to think of identity in terms of the characterisation questions, she also thinks that characterisation is key for answering philosophical questions such as why people care about future pleasure or pain or how we attribute moral responsibility for past actions. For Schechtman, such questions are about qualitative rather than numerical identity to past and future selves. She thinks that the answer is to adopt a narrative theory. According to Schectman, persons constitute their identities by organising their experiences into continuing narratives about their lives, which incorporate the experiences (or anticipated experiences) of their past and future selves. The characteristics that make up an individual's identity are those that cohere together in a narrative structure (see also MacIntyre^16^).

The characterization theory is more than merely a descriptive claim that people weave their lives into coherent stories (on which see, eg, other works from Bruner, Dennett and Sacks^22^, ^23^, ^24^). What Schectman adds is the argument that narrative coherence is the relevant criterion for various practical concerns. However, we might wonder why people's subjective narratives about their lives should be the basis of what look like objective claims, such as whether they are morally responsible for their past actions. This is doubly true when we apprehend that the telling of a story often involves a reinterpretation of the facts to make a more coherent narrative and that some people's subjective stories may end up being very far from the truth of their lives. (See Schechtman^25^ for some attempts to counter this objection). ‡
Worries about the status of reinterpretations also emerge in debates about whether memory is a reliable epistemological source. Memory retrieval involves reconstruction and simulation. But memory is still reasonably accurate on average and does not need to be exactly right to be functional.^26^



However, even at the descriptive level, it has been argued that the narrative account is not sufficient for self. Zahavi argues that it does not capture the phenomenology of our distinctive first‐person perspective on experiences.^27^, ^28^ If we strip away all narrative and self‐construction, there is still a subjective sense of the ownership of our experiences that exists prior to any reflection. Hence, Zahavi claims that to make sense of a first‐person perspective, we already need to invoke a self. He calls this immediate subject of experience the “minimal self.” The minimal self is not extended in time, although it can experience the passing of time, and it does not have a narrative structure. Therefore, for Zahavi, the narrative self presupposes the minimal self but not vice versa.

Strawson goes further and argues that narratives are not necessary for either a descriptive or a normative theory of self.^29^ He allows that some people may be *diachronics*, who experience themselves as persisting into the past and the future, but argues that others (such as himself) are *episodics*, who experience their self as existing solely in the present. Even if their existence as an experiencer is fleeting, episodics may know, as a fact, that they are part of a continuing human being (which may persist according to biological continuity views, therefore, they could accept that they are numerically identical with the human being according to those views). However, if episodics consider that they will not exist in the past and the future, then they may have no special concern for their past or any tendency to view their lives in a narrative form. On a normative level, Strawson thinks that this can still be a valuable way of living a life.

3 Personhood and agency

Another objection to persistence theories is that they take the wrong approach to answering the motivating questions about egoistic, moral, and compensatory concern because the answers require a prior conception of agency or personhood. This criticism takes 2 different forms, but both imply that being a diachronic agent is what matters. In this context, philosophers use “agent” to mean the entity that takes actions, which is different from the phenomenological feeling of self‐ownership of those actions.

We can distinguish 2 sorts of arguments for the primacy of agency: that it is diachronic agency rather than the metaphysics of personal identity that matters^30^ and that our metaphysical theories of personal identity depend on a conception of the person, which requires a prior account of agency.^31^, ^32^ In both cases, the idea of agency already has an intertemporal aspect built into it.

Korsgaard argues that metaphysics is not the right foundation for personhood and criticizes persistence accounts for thinking of the self as a locus of experience, rather than as a doer.^30^ She inverts their order of explanation. Instead of your leading one continuing life because you are one person, she argues the reverse: that you are one continuing person because you have one life to lead. Many of our projects extend over time, and some of our ultimate ends, like preserving our health, presuppose an ongoing identity. Choosing amongst actions already takes us some way into the future, as actions are temporally extended; to the extent that you regulate your choices because you see yourself as implementing a life plan, you are already identifying with yourself in the future, as a part of how you see yourself now. Our practical projects give us a reason for regarding ourselves as being the same person as the self who will occupy our body in the future.

For Korsgaard, viewing oneself as an intertemporal unity is a practical requirement of being an agent, regardless of the metaphysics.^30^ For others, the intertemporal nature of agency is a part of the metaphysics.^31^, ^32^ For instance, Rovane argues that the metaphysics of personhood is intrinsically normative, so we should start by specifying an “ethical criterion of personhood”^32^ and use that to determine what entities count as persons. Rovane's chosen criterion is rational agency, which involves a commitment to achieving overall *rational unity*, such that a person's earlier commitments will govern their later actions.

Both arguments for the primacy of agency connect agency to rationality and reasons. Therefore, coherence and stability over time are built into the idea of the person as an agent. §
Some people who take the agency approach also argue that there can be agents that are either greater or lesser than the individual.^32^, ^33^ One consequence of Rovane's position is that groups may be agents, so long as they are committed to achieving rational unity. Another is that there can be 2 agents in one human being where there are distinct streams of rational unity, for instance, in cases of multiple personality/dissociative identity disorder. Radden, based on cases of extreme self‐fragmentation found in psychopathology, argues that “if our subselves are construed more as agents than as the subjects of phenomenological states, it seems possible to accept their being units smaller than body.”^34^



## 3 PHILOSOPHICAL INSIGHTS INTO SELF OVER TIME AND THE FRAMEWORK OF A PERSON AS AN INTRAPERSONAL TEAM REASONER

From these 3 philosophical approaches, we can distinguish 3 ways in which a person can be a self over time, as an experiencer, a story teller, and a doer, or the experiential, narrative, and agentic selves. These correspond to 3 different ways someone can relate to his or her self at a different time, which we might call the relationship between different “synchronic selves.” We will avoid being too specific about the duration of a synchronic self. In this, we follow Prebble, who allows that the unit of time comprising the present can vary depending on the aspect of the self under investigation.^35^ We explain more below.

The self as experiencer is drawn from psychological continuity accounts of persistence, from which we can extract the idea of self over time as a matter of connectedness. The term “experiencer” derives from Korsgaard's observation that psychological continuity theories take the self to be a locus of experience.^30^ The experiential self is not to be confused with the minimal self of Zahavi.^27^, ^28^ The minimal self is pre‐reflective, whereas the experiential self includes the possibility that the self has conceptual content and is not a phenomenological notion. The minimal self is restricted to James' *I‐self*, the subject of experiences, whereas the experiential self is his *me‐self*, the object of this awareness, a person's mental representation or model of her self (her “self‐concept”), which comprises all the things that she perceives and knows about herself and which has semantic content.^36^ The I‐self is fleeting and prior to any reflection, although it has the capacity for reflection and the experience of reflecting. The me‐self includes the content of reflective thought at the present moment, but it may also extend over a period in which there is no significant change in the self‐concept.^35^ ¶
Some philosophers might prefer to think of the idea that the me‐self can be extended over time as merely a convenient linguistic shorthand for an aggregate of “timeslices,” the momentary selves that are found in metaphysics (which are different from the minimal self as they are not restricted to phenomenological content), over a period when the timeslices are all qualitatively the same.


The experiential self may perceive that it is connected to past and future selves, in the sense of Parfit discussed above, which may engender a subjective feeling of connectedness with other synchronic selves. Hence, the perception of connectedness may give rise to a phenomenology of continuity, a feeling that one extends through time, which is nevertheless a feeling that is experienced at a time and therefore could be had by a minimal self (Kennet and Matthews talk about “a distinct feeling of there being a continuous experiential ‘worm’” connecting the synchronic selves^37^). This is similar to an idea found in the writing of James, who thought that the current self's perception that it is similar to proximate selves gives rise to a sense that the current self is continuous with those proximate selves.^36^

The self as a storyteller derives from the narrative account of self. The narrative self generates diachronicity through the ability of a synchronic self to connect life events to the present me‐self. The synchronic self becomes an author who constructs her self over time. She is the same person, despite changing over time, provided that she can make those changes intelligible to herself. The self as storyteller leaves both the subjective and agential features of self unaddressed. Indeed, even though she is a proponent of the narrative self, Schechtman has argued that her theory needs to be supplemented by an account of “empathic access,” roughly the ability to recall past emotions, thoughts, and feelings combined with a sympathetic attitude towards the states recalled.^38^

The narrative self has been influential in theorizing about the self in BPD.^9^, ^39^ The impairments described in DSM‐5 have been viewed by some in terms of defective narrative structures or narrative sequences. This is most easily seen with regard to identity, where it has been argued that a poorly developed self‐image corresponds to incoherent narrative structures^39^ and the unstable sense of self corresponds to discontinuous narrative sequences.^40^ However, the appeal to narratives has also extended to self‐directedness, with the idea that one makes sense of the acting subject within a narrative framework that includes, eg, aspirations, values, and career plans.^39^ The narrative approach is also central to clinical practice in psychiatry, where the encouragement of storytelling and the subsequent assessment of event‐scripts forms a major part of history‐taking, clinical formulation (the theoretical explanation of clinical phenomena used in diagnosis), engagement (patient involvement in treatment), and of several aspects of psychotherapy.^41^ Therapeutic interventions based on narrative have been used to give people a phenomenological sense of continuity, including in the treatment of BPD patients.^42^, ^43^ Dimaggio et al have also proposed that facilitating the formation of autobiographical memories can enrich the narrative self of subjects with personality disorder, leading to a more stable sense of identity.^44^

However, if Strawson^29^ is right that the nonclinical population can be nonnarrative, that challenges the idea that lack of narrative causes the pathology in BPD. It is necessary to articulate the sense in which self and identity is pathological in BPD. We suggest that the answer may be found in a different conception of self, the agentic self.

The self as doer, or agentic self, is derived from theories of personhood and agency. Here, the source of self over time is the unity imposed on synchronic selves who see themselves as a single agent over time. The emphasis is on the conception of self as a temporal entity, with a personal past, present, and future. Rather than starting with a synchronic self that extends itself over time, we prioritise the diachronic self. This is consistent with the intuitive idea that a person who has a deficit in diachronic self, for instance, rapid and frequent changes in values and life goals, may have problems in ascribing themselves a coherent synchronic self.

The recognition that synchronic selves are all part of the same diachronic agent allows the current synchronic self to “identify” with past and future selves. Identification is the recognition that a self in the past or the future was or will be part of the same person. We can clarify this with a metaphor from James. The owner of a herd of cattle can recognise his animals because they are branded. However, “no beast would be so branded unless he belonged to the owner of the herd. They are not his because they are branded; they are branded because they are his.”^36^ The selves at different times, which are equivalent to the cattle in the metaphor, can recognise that they are branded the same; being branded the same derives from being a part of the same entity over time.

The semantic understanding that an action or experience was or will be mine is typically accompanied by a feeling of “mine‐ness,” as a person projects herself into the past and the future. This relates to “autonoetic consciousness,” which provides a recollective experience infused with a sense of one's self extended in time, which we define and discuss in more detail below.^45^, ^46^

Although connectedness and identification tend to cooccur, they are conceptually different and they can come apart.^37^, ^47^, ^48^ Identification can occur even in the absence of connectedness or a feeling of cognitive continuity. It can unify synchronic selves that are very far apart in time, which may be very dissimilar, and which the current synchronic self may not feel connected to.

We can demonstrate this clearly using a model from decision theory. In decision theory, when an individual has to make a series of choices over time, it is standard to model the choice as being made by a series of “transient agents,” who are the loci of experience and who make choices. Each transient agent has its own preferences, and, although some preferences may be held in common, there may also be conflicts of interest. For example, the transient agents might all prefer that some unpleasant activity be undertaken, such as writing a report, but they may have differing preferences about which transient agent should do it. Standard decision theoretic reasoning allows each transient agent to ask “What do I, as a synchronic self, want and what should the present self do to achieve it?”. The transient agents are modelled as experiencing selves. When there are conflicts of interest between the preferences of transient agents, this reasoning can lead to procrastination, the breaking of resolutions, and the failure to implement long‐term plans.^48^, ^49^, ^50^

In situations with conflicts of interest, there are 2 different ways that the transient agents in the model can achieve their long‐term plans, which correspond to connectedness and identification.

They may recognise that they are connected to transient agents in other periods, leading them to be concerned about the outcomes that will be experienced in other periods—a concern that then gets incorporated into their preferences. This may reduce the conflict between the transient agents' preferences, increasing the likelihood that they can carry through long‐term plans. However, it is natural to think that people place more weight on the present than the future,^51^ so connectedness alone may not be enough to resolve conflicts.^48^, ^50^

Alternatively, the transient agents may identify with the person over time, which is an agent that exists over time and pursues courses of action that unfold over time, like the agent in the agentic theory of the self. The person over time is composed of a “team” of transient agents. When they identify with the team, the transient agents can use *intrapersonal team reasoning*, a model of reasoning that allows them to ask, “What do I, as a person (team) over time, want and what actions should the present self take to achieve it?”.^47^, ^48^, ^50^, ^52^ Identifying with the team unifies the transient agents, regardless of how connected they are with their past and future, or of their concern for the other timeslices. It offers a sense of self that may be had by people who are not narrative but also not pathological, like Strawson, and explains how they can act on past commitments, pursue long‐term plans and generally live successful lives.

In the usual case, we would expect a feedback loop between identification and connectedness: Identifying with your past and future synchronic selves should also increase your sense of being connected to them. But identification and connectedness are separate processes, and it is possible to find cases where only one occurs.^37^

The model of self as an intrapersonal team reasoner addresses agential elements of self. It includes both a present experiencer (the transient agent) and an agent—or person—over time (the team of transient agents), who acts in pursuit of projects that extend over time. It shows how experiencers can relate to an agentic self over time and hence to the person's other experiential selves, via identification, which need not involve any recourse to narratives about their own lives. This is not to say that narratives are not helpful—some types of narrative may boost the sense of continuity or identity between transient agents—but identification can be done even in the absence of narrative self.

In addition, the model makes it clear how agentic accounts of self can connect the unstable sense of self to another of the BPD diagnostic criteria, that of disinhibition.^47^ In the DSM, disinhibition, which is a broad, higher‐order personality trait, is characterized by irresponsibility, or a disregard for and failure to honour commitments, agreements, and promises; impulsivity, or acting on the spur of the moment without consideration of outcomes, and difficulty establishing and following plans; risk taking, unnecessary engagement in dangerous and potentially self‐damaging activities, without regard for consequences.^7^ Lack of identification and lack of a sense of continuity can both lead someone to act on fleeting preferences, rather than on a long‐term coherent plan. Conversely, either feeling connected to one's future self or identifying with the future self may make one more likely to consider the consequences of one's actions and to act on stable values and goals. Psychologists have found that feeling connected to one's future self may make one more likely to make sacrifices now for the sake of long‐term goals.^53^, ^54^ A relationship between identification and self‐control has been found in the context of mental time travel, which we will explain below.

## 4 MEMORY AND SELF‐PATHOLOGY IN BPD

Philosophy of personal identity traditionally thinks of memory as producing a diachronic self.^10^ However, the relationship between memory and selfhood is more complicated than that. The self is not only manifest in memory but also in emotions, perceptions, and actions, elements of which can figure in the act of remembering, so connections between self and memory can run in both directions. We review 2 rather different connections between memory and self, which are both associated with the pathology of BPD, and relate them to the agentic account of self.

### 4.1 Episodic memory and mental time travel

Autobiographical memory is a complex mental system that allows people to recollect information, events, and experiences from their pasts.^35^, ^55^ It involves both semantic and episodic memories. Semantic memory is the recall of facts and propositions; episodic memory is the recall of an experienced episode. There is a phenomenological difference because retrieving semantic memory feels like knowing, whereas retrieving episodic memory typically involves reliving or reexperiencing. The sense of mine‐ness that typically attaches to episodic memories is called *autonoetic consciousness*,^56^ which allows the I‐self to feel that it has existed in the past or will exist in the future.^35^ We suggest that the unstable sense of self in BPD may be associated with disruptions in this temporally extended I‐self.

For Tulving, the phenomenology of autonoetic consciousness is a part of the definition of episodic memory and one of the functions of episodic memory is to make mental time travel possible.^57^ *Mental time travel* involves imaginatively projecting oneself into a mentally simulated event, either in the past or in the future.^26^, ^56^, ^58^ Remembering past episodes and imagining the future use some of the same skills and brain circuitry,^59^, ^60^ in particular, scene construction and self‐projection through time.^61^, ^62^ *Scene construction* is the process of mentally generating and maintaining a complex and coherent scene or event.^63^ *Self‐projection* is the ability to shift perspectives away from the immediate present.^64^ Autonoetic consciousness is a type of self‐projection.^65^ For Tulving, autonoetic consciousness is a necessary correlate of episodic memory.^56^ The phenomenology of ownership of the experience is a part of his definition of episodic memory; scene reconstruction without self‐projection would not count as episodic memory.

Prebble and colleagues argue that the minimal self is necessary for the capacity for self‐awareness, which is considered “a vital precursor to autonoetic consciousness and episodic memory” because self‐awareness is “necessary to differentiate a mental state that is ‘remembered’.”^35^ They survey evidence that lack of pre‐reflective self‐awareness causes deficits in autonoetic consciousness.

Neurological evidence also suggests a connection between self‐projection and pre‐reflective self‐processing. The capacity for self‐projection is supported by a core brain network, which is activated in resting states.^64^ There is a neural overlap between this resting state brain activity and general self‐referential processing, or the processing of items and stimuli related to oneself.^66^ The brain areas involved in self‐projection overlap with the cortical midline structures, which are involved in self‐referential processing. Hence, it is not surprising that self‐referential processing deficits, which have been studied in psychiatric disorders, can lead to changes in forms of self‐projection, such as autonoetic consciousness.^35^

Neuroimaging studies of BPD patients have shown that they have altered self‐processing.^67^ Further work is needed to explore the specific forms of and deficits in self‐projection involved in BPD. We speculate that the unstable sense of self is related to a disruption of the temporally extended subjective sense of self, a function that is thought to be supported by mental time travel.

Mental time travel also connects an impaired sense of self to disinhibition. Mental time travel goes forwards in time, as well as backwards, and mental time travel into the future may be a prerequisite for rational agency.^37^, ^68^ Autonoetic consciousness allows us to pre‐experience the future consequences of our actions, which can help us to delay rewards and make choices that will be more advantageous in the long‐term.^69^, ^70^, ^71^ Individuals who have a predisposition to be less concerned about the future consequences of their actions benefit most from imagining future scenarios.^70^ Impaired autonoetic consciousness leads to problems with planning, prospective memory, episodic future thinking, and general thinking about one's future.^72^

### 4.2 Autobiographical memory and dissociation

Autobiographical remembering also involves forms of processing that enable the transformation of raw, experiential, and subjective material to semanticized memories. Representing our self across time in this manner gives temporal extension to the me‐self.^35^ Borderline personality disorder patients have deficits in autobiographical memory. These are sometimes explained using a narrative account of self, focusing on the semantic contents of memory retrieval. We will show that the deficits are also consistent with an agentic account.

Borderline personality disorder patients have abnormal autobiographical memory functioning; for instance, they show memory biases in autobiographical recall.^73^ Their memory deficits have been connected to problems with their narrative selves. Memory problems have been examined in the context of dissociative experiences, where the subjects may have large gaps in their memories, may be bothered by how much they have forgotten and may not remember what they did or said when angry.^74^, ^75^ However, dissociation is also associated with behavioural dyscontrol,^76^ which suggests that the memory deficits can also be explained by processes related to agency.

Borderline personality disorder patients often have mild and fluctuating dissociation, which Meares (borrowing from Janet) calls “continuous forgetfulness.”^77^ Dissociation involves the disruption of consciousness, memory, identity, or perception of the environment. Dissociation ranges along a spectrum ranging from the nonpathological, such as daydreaming, to the pathological, such as dissociative amnesia, “amnesia for personal information or events that are too extensive to be explained by ordinary forgetfulness.”^75^ In BPD, dissociation is often pathological, with dissociative amnesia leading to characteristic mental holes or blind spots in patients' autobiographical memories. There is abundant evidence that susceptibility to dissociation is connected to having experienced traumatic events and adverse early environments.^78^

Dissociation has been connected to the narrative self. Bessel Van Der Kolk^79^ writes that “traumatic memories are fundamentally different from the stories we tell about the past. They are dissociated: The different sensations that entered the brain at the time of the trauma are not properly assembled into a story, a piece of autobiography.” However, he also stresses that the narrative self, which is rooted in language, cannot substitute for the moment‐to‐moment sense of self, which is based primarily on physical sensations and emotions, resulting in action.

Another psychological phenomenon that has been thought to undermine the coherence of the autobiographical narrative of BPD patients is the formation of overgeneral memories. People are said to have *overgeneral memory* when they tend to recall categories of events, rather than specific memories, even when explicitly instructed to recall specific events. Overgeneral memory is thought to be a coping strategy that avoids the retrieval of specific painful memories. Jones and colleagues found that patients suffering from BPD had difficulty in recapturing specific personal memories, particularly relating to negative events.^80^ (Although for a critical appraisal that disputes the phenomenon, see Van den Broeck et al^81^) Meares hypothesizes that BPD patients will display overgeneral memory as a way of avoiding traumatic memories and that this lack of specific memories also precludes patients from forming coherent self‐narratives.^9^

However, it is also possible that dissociation and memory problems result from agentic deficiencies. The same processing styles that cause behavioural disinhibition can cause instability of self, if they are applied to self‐relevant information. Berzonsky categorizes people according to their identity processing styles.^82^, ^83^ The way that people process self‐relevant information is related to their approach to personal decision making and problem solving. The *avoidant‐diffuse* identity type is characterized by impulsive and ad hoc problem solving, focusing on the short‐term, and generally postponing decisions “until situational consequences and rewards dictate a course of action.”^82^ Borderline personality disorder patients are predominantly avoidant‐diffuse.^84^ Their processing of self‐relevant information is influenced by impulsivity, emotion‐focused coping strategies, and tendencies to rely on an external locus of control. Hence, the sort of processing deficits that causes disinhibition may also manifest as self‐processing deficits, which cause the characteristic mental holes or blind spots that can figure in the autobiographical memories of BPD patients.

## 5 CONCLUSION

The agentic self provides a novel theoretical framework for research on identity disturbance in BPD, which complements the narrative approach. We used the decision theoretic model of the self over time as an intrapersonal team reasoner to clarify features of the agentic self. Agency contrasts with connectedness and unifies the self over time in a way that is compatible with being nonnarrative. The intimate connection between diachronic agency and actions over time provides a theoretical basis for the cooccurrence of unstable sense of self and disinhibition in BPD patients.

The agentic approach illuminates diachronic identity and self‐directedness. Identity disturbance in BPD has both diachronic and synchronic elements. The 2 types of deficit may be related. If someone has constantly changing values and life goals, then it may not be surprising that they do not have a coherent synchronic self. The model of the agentic self suggests the potential for investigating the construct of self instability by focusing on temporal aspects of self and mental time travel.

In sum, the agential self is a promising addition to the literature on BPD. In this short paper, we cannot do justice to the many connections between agency, action over time, and self. The agentic approach to BPD merits further research.
